# Effect of Computer-Assisted Cognitive Behavior Therapy vs Usual Care on Depression Among Adults in Primary Care: A Randomized Clinical Trial

**DOI:** 10.1001/jamanetworkopen.2021.46716

**Published:** 2022-02-10

**Authors:** Jesse H. Wright, Jesse Owen, Tracy D. Eells, Becky Antle, Laura B. Bishop, Renee Girdler, Lesley M. Harris, R. Brent Wright, Michael J. Wells, Rangaraj Gopalraj, Michael E. Pendleton, Shehzad Ali

**Affiliations:** 1 Department of Psychiatry and Behavioral Sciences, University of Louisville School of Medicine, Louisville, Kentucky; 2 Department of Counseling Psychology, University of Denver, Denver, Colorado; 3 Kent School of Social Work, University of Louisville, Louisville, Kentucky; 4 Department of Medicine, University of Louisville School of Medicine, Louisville, Kentucky; 5 Department of Family and Geriatric Medicine, University of Louisville School of Medicine, Louisville, Kentucky; 6 now with Norton Healthcare, Norton Community Medical Associates, Louisville, Kentucky; 7 now with Baptist Health, Baptist Health Medical Group Primary Care, Louisville, Kentucky; 8 Mental Health and Addictions Research Group, Department of Health Sciences, University of York, Heslington, United Kingdom; 9 Department of Psychology, Macquarie University, Sydney, New South Wales, Australia; 10 Department of Epidemiology and Biostatistics, Schulich School of Medicine, Western University, London, Ontario, Canada

## Abstract

**Importance:**

Depression is a common disorder that may go untreated or receive suboptimal care in primary care settings. Computer-assisted cognitive behavior therapy (CCBT) has been proposed as a method for improving access to effective psychotherapy, reducing cost, and increasing the convenience and efficiency of treatment for depression.

**Objectives:**

To evaluate whether clinician-supported CCBT is more effective than treatment as usual (TAU) in primary care patients with depression and to examine the feasibility and implementation of CCBT in a primary care population with substantial numbers of patients with low income, limited internet access, and low levels of educational attainment.

**Design, Setting, and Participants:**

This randomized clinical trial included adult primary care patients from clinical practices at the University of Louisville who scored 10 or greater on the Patient Health Questionnaire–9 (PHQ-9) and were randomly assigned to CCBT or TAU for 12 weeks of active treatment. Follow-up assessments were conducted 3 and 6 months after treatment completion. Enrollment occurred from June 24, 2016, to May 13, 2019. The last follow-up assessment was conducted on January 30, 2020.

**Interventions:**

CCBT included use of the 9-lesson computer program Good Days Ahead, along with as many as 12 weekly telephonic support sessions of approximately 20 minutes with a master’s level therapist, in addition to TAU, which consisted of the standard clinical management procedures at the primary care sites. TAU was uncontrolled, but use of antidepressants and psychotherapy other than CCBT was recorded.

**Main Outcomes and Measures:**

The primary outcome measure (PHQ-9) and secondary outcome measures (Automatic Thoughts Questionnaire for negative cognitions, Generalized Anxiety Disorder–7, and the Satisfaction with Life Scale for quality of life) were administered at baseline, 12 weeks, and 3 and 6 months after treatment completion. Satisfaction with treatment was assessed with the Client Satisfaction Questionnaire–8.

**Results:**

The sample of 175 patients was predominately female (147 of 174 [84.5%]) and had a high proportion of individuals who identified as racial and ethnic minority groups (African American, 44 of 162 patients who reported [27.2%]; American Indian or Alaska Native, 2 [1.2%]; Hispanic, 4 [2.5%]; multiracial, 14 [8.6%]). An annual income of less than $30 000 was reported by 88 of 143 patients (61.5%). Overall, 95 patients (54.3%) were randomly assigned to CCBT and 80 (45.7%) to TAU. Dropout rates were 22.1% for CCBT (21 patients) and 30.0% for TAU (24 patients). An intent-to-treat analysis found that CCBT led to significantly greater improvement in PHQ-9 scores than TAU at posttreatment (mean difference, −2.5; 95% CI, −4.5 to −0.8; *P* = .005) and 3 month (mean difference, −2.3; 95% CI, −4.5 to −0.8; *P* = .006) and 6 month (mean difference, −3.2; 95% CI, −4.5 to −0.8; *P* = .007) follow-up points. Posttreatment response and remission rates were also significantly higher for CCBT (response, 58.4% [95% CI, 46.4-70.4%]; remission, 27.3% [95% CI, 16.4%-38.2%]) than TAU (response, 33.1% [95% CI, 20.7%-45.5%]; remission, 12.0% [95% CI, 3.3%- 20.7%]).

**Conclusions and Relevance:**

In this randomized clinical trial, CCBT was found to have significantly greater effects on depressive symptoms than TAU in primary care patients with depression. Because the study population included people with lower income and lack of internet access who typically have been underrepresented or not included in earlier investigations of CCBT, results suggest that this form of treatment can be acceptable and useful in diverse primary care settings. Additional studies with larger samples are needed to address implementation procedures that could enhance the effectiveness of CCBT and to examine potential factors associated with treatment outcome.

**Trial Registration:**

ClinicalTrials.gov Identifier: NCT02700009

## Key Points

**Question:**

Does computer-assisted cognitive behavior therapy (CCBT) plus treatment as usual (TAU), compared with TAU alone, improve treatment outcome for depression in primary care patients?

**Findings:**

In this randomized clinical trial of 175 adults, CCBT reduced depression, as measured by the Patient Health Questionnaire–9, to a significantly greater extent than TAU and was associated with remission rates that were more than double those observed for TAU.

**Meaning:**

In this study, CCBT was an efficacious way to treat depression as well as increase access to evidence-based psychotherapy for primary care patients.

## Teaser

This randomized clinical trial evaluates whether clinician-supported computer-assisted cognitive behavior therapy (CCBT) is more effective than treatment as usual in primary care patients with depression, with a focus on primary care patients from minoritized groups and those with low income.

## Introduction

Depression is a common disorder that is often undertreated.^1,2,3,4^ The annual prevalence rate for major depressive disorder (MDD) is approximately 10.0% to 12.5% in primary care populations.^2,3^ Although collaborative care has been shown to improve treatment in primary care,^2,5,6^ problems remain in providing evidence-based therapies for depression.^7,8,9^ Multiple barriers to receiving psychotherapy have been described, including cost and time constraints.^7^

Computer-assisted cognitive behavior therapy (CCBT) has potential for providing effective psychotherapy in primary care.^9,10,11,12,13,14,15,16,17,18^ In CCBT, a computer program is used for building CBT knowledge and skills, thus reducing the amount of clinician time needed for treatment. Some potential advantages for CCBT identified by researchers are reduced cost and enhanced access to treatment. Multiple meta-analyses have found that CCBT is associated with improved depressive symptoms if the computerized elements of treatment are partnered with clinician support.^19,20,21,22,23,24,25,26,27,28,29^ A meta-analysis of CCBT for depression that included only studies in primary care^18^ suggested that CCBT may have somewhat weaker associations with improved depression in primary practices than other settings.^18^ However, the number of studies in primary care was small (n = 8), and the reasons for possible disparities in outcomes were unclear. Concerns regarding previous CCBT research include potential bias of recruitment strategies (eg, online advertisements, requirement for internet access) that preferentially select those with high levels of education and computer skills, limited inclusion of persons with lower income and lack of internet access, and insufficient attention to implementation issues.^29,30,31,32^

The objectives of the current investigation were to (1) evaluate the effectiveness of CCBT compared with treatment as usual (TAU) in primary care patients with depression; (2) evaluate the feasibility of CCBT in a primary care population with substantial numbers of patients with low levels of education, reading skills, and/or internet access: (3) perform an exploratory analysis of factors associated with differential treatment outcome; and (4) evaluate the medical care utilization costs of CCBT vs TAU. The economic analysis results will be reported separately.

## Methods

### Study Design, Setting, and Participants

In this randomized clinical trial, participants were recruited by referral from the clinical practices of the Departments of Family and Geriatric Medicine and Internal Medicine at the University of Louisville. All clinics were in urban settings in Louisville, Kentucky, except for 1 rural family medicine site in Glasgow, Kentucky. Enrollment occurred from June 24, 2016, to May 13, 2019. The last follow-up assessment was conducted on January 30, 2020, when the trial ended as planned. Eligibility screening was conducted by a research associate who administered the Patient Health Questionnaire–9 (PHQ-9),^33^ Columbia Suicide Severity Rating Scale (CSSRS),^34^ and a brief exclusion criteria questionnaire. If patients met exclusion criteria not requiring an evaluation with the Mini International Diagnostic Interview (MINI),^35^ they were not assessed further with the MINI and the reading subtest of the Wide Range Achievement Test (WRAT).^36^ Exclusion criteria were (1) PHQ-9 score less than 10; (2) refusal to provide informed consent; (3) aged 17 years or younger; 4) self-report of inability to read English; (5) significant suicidal risk found on CSSRS (Antle et al^32^); (6) severe medical disorders that would interfere with CCBT (eg, liver failure, terminal cancer); (7) dementia or other organic brain disorders; and (8) MINI diagnosis of psychotic disorder or bipolar disorder. During the first 6 months of recruitment, patients were excluded for scoring less than a ninth-grade level on the WRAT reading test. However, this exclusion was removed after more patients than expected (3 of 19) who otherwise desired to participate were denied entry to the study. All participants provided written informed consent. The study was approved and monitored by the University of Louisville institutional review board. Study methods followed Consolidated Standards of Reporting Trials (CONSORT) reporting guideline. The trial protocol appears in Supplement 1.

### Interventions

CCBT was provided for 12 weeks with use of the 9-lesson Good Days Ahead (GDA) computer program^33,34,35,36,37,38,39,40^ and TAU plus as many as 12 telephonic support sessions with a master’s level mental health clinician. CCBT was an add-on treatment to TAU. Text or email communications, based on patient preference, were permitted with the clinician supporting CCBT. The design specified an average of 20 minutes total support time per week. Actual time spent in each support activity was recorded. CCBT methods with GDA are described in detail elsewhere.^32,38,39^ Treatment as usual (TAU) included the standard clinical care by physicians in the primary care practices. TAU was uncontrolled, but use of antidepressants and other psychotherapies were recorded.

Because 42 of 141 patients (29.8%) surveyed in the University of Louisville primary care setting before the study began had no internet access, the study design included the loan of low-cost laptops with internet access (total cost per device, $253) to those who requested them (17 of 175 study patients [9.7%]).

### Outcome Measures

The primary outcome measure was the PHQ-9,^33^ a widely used tool for assessing depression. Secondary outcome measures were the Automatic Thoughts Questionnaire (ATQ)^41^ for negative cognitions, the Generalized Anxiety Disorder–7 (GAD-7),^42^ and the Satisfaction with Life Scale (SWLS)^43^ for quality of life. Each were administered before treatment, after 12 weeks (ie, end of treatment with CCBT), and at 3 and 6 months after treatment. The Client Satisfaction Questionnaire–8 was administered at the posttreatment and 3- and 6-month follow-ups.^44^ Rating scales were completed online unless patients requested they be done by telephone.

### Randomization

Randomization was conducted by the research associate after consent to study participation and completion of screening assessments, including the MINI and the baseline measures. Each participant was randomly assigned to CCBT or TAU without stratification in real time for each patient through a random number generator instead of setting up a randomized assignment for the target sample size prior to initiating the study.

### Treatment Completion and Fidelity

Prior to the start of the study, we specified criteria for defining dropout and completer status (eAppendix in Supplement 2). Completer status for CCBT was defined as completing at least two-thirds of the therapy content (6 of 9 lessons in GDA and 9 of 12 telephone or email sessions with the therapist). Noncompleters of CCBT were not considered dropouts if they continued in the study and completed measures. The clinician providing therapeutic support was supervised approximately twice monthly by 1 of us (J.H.W.) using audio recordings of treatment sessions. Fidelity was assessed with the Cognitive Therapy Rating Scale (CTRS).^45,46^ Of 40 sessions rated on the CTRS, the mean (SD) score was 55.23 (5.32) with a range of 43 to 62. The highest possible score on the CTRS is 66. A score of 40 is commonly used to indicate adequate fidelity to CBT methods. The treating clinician had prior experience performing CBT but no experience in CCBT.

### Adverse Events

A data safety monitoring board monitored the integrity of the study on an annual basis. Adverse events were reported to them and the study sponsor (Agency for Healthcare Research and Quality).

### Statistical Analysis

#### Sample Size

An a priori power analysis found that a sample size of 98 participants per group (N = 196) would be sufficient to detect a medium-sized effect (Cohen *d* of approximately 0.50) with 80% power. These estimates were based on previous research examining active treatments vs TAU^47^ and a previous meta-analysis on CCBT.^29^ Because the sample size projection considered an anticipated 20% dropout rate, the target sample size was 240 patients.

#### Statistical Testing

The intent-to-treat (ITT) analyses and multiple imputation analyses were performed with MPlus version 7.3 (Muthén & Muthén), and SPSS version 25 (IBM Corp) was used for other descriptive and baseline comparisons. Growth curve models with random effects were conducted for the primary outcome variable (PHQ-9) and secondary outcomes. These models use a multivariate approach in which residuals were allowed to vary and the parameters to correlate. A quadratic change model was constructed (ie, linear growth until 3 months and then leveling off) with treatment condition estimating intercept and slopes. Two-tailed tests were used at a .05 level of significance. Multiple imputation was used, with 20 imputed data sets for the primary and secondary outcomes. To create the data sets, the treatment condition was included along with the variables at each point (eg, PHQ-9 score). Parameters and standard errors were averaged based on the Rubin approach.^48^ Calculations were also performed on how many patients responded to treatment (defined as a 50% reduction in PHQ-9 scores from pretreatment to posttreatment) and reached remission from depression (defined as a posttreatment score of less than 5 on the PHQ-9).^49^

#### Missing Data

To explore whether the degree of missing data was associated with treatment condition and outcome, we applied methods of Enders^50^ and Wu and Carroll^51^ that are tailored to conducting growth curve models. A polynominal regression was used to predict patterns of missingness (ie, complete, intermittent, termination) associated with treatment condition, baseline depressive symptoms, or the change in depressive symptoms.

## Results

### Participant Characteristics

The sample of 175 patients was predominately female (147 of 174 who reported [84.5%]) and had a high proportion of racial and ethnic minorities (African American, 44 of 162 patients who reported [27.2%]; American Indian or Alaska Native, 2 [1.2%]; Hispanic, 4 [2.5%]; multiracial, 14 [8.6%]). The mean (SD) age was 47.03 (13.15) years; 129 of 173 patients who reported (74.6%) had less than a college education; and 88 of 143 patients (61.5%) reported an annual income of less than $30 000 (Table 1). There were no significant baseline differences in the PHQ-9, ATQ, GAD-7, and SWLS scores. Reading proficiency measured by the WRAT was comparable in both groups. The range of reading levels in the entire sample was grade 1.8 to 13.0 (mean [SD], 11.7 [2.2]). Nineteen patients (10.9%) had reading levels lower than ninth grade. Completion and dropout data are shown in the study flowchart (Figure).

**Table 1.** Baseline Demographic Characteristics and Diagnoses for Patients Receiving CCBT and TAU

Characteristic	Patients, No./total No. (%)	
	CCBT	TAU
Sex		
Women	76/94 (80.9)	71/80 (88.8)
Men	18/94 (19.1)	9/80 (11.3)
Race and ethnicity		
African American	22/85 (25.9)	22/77 (28.6)
American Indian or Alaska Native	1/85 (1.2)	1/77 (1.3)
Hispanic	2/85 (2.4)	2/77 (2.6)
White	52/85 (61.2)	46/77 (59.7)
Multiracial or multiethnic	8/85 (9.4)	6/77 (7.8)
Current psychotherapy^a^		
Yes	24/93 (25.8)	25/79 (31.6)
No	69/93 (74.2)	54/79 (68.4)
Current antidepressant^b^		
Yes	22/94 (23.4)	19/79 (24.1)
No	72/94 (76.6)	60/79 (75.9)
Annual income, $		
0-14 999	35/72 (48.6)	29/71 (40.8)
15 000-29 999	7/72 (9.7)	17/71 (23.9)
30 000-44 999	11/72 (15.3)	10/71 (14.1)
45 000-59 999	5/72 (6.9)	5/71 (7)
60 000-74 999	5/72 (6.9)	4/71 (5.6)
≥75 000	9/72 (12.5)	6/71 (8.5)
Primary psychiatric diagnosis		
Major depression	76/95 (80)	69/80 (86.3)
Anxiety disorder	2/95 (2.1)	0/80
None	17/95 (17.9)	11/80 (13.8)
Education		
Eighth grade	7/93 (7.5)	4/80 (5)
High school graduate	57/93 (61.3)	61/80 (76.3)
College graduate	29/93 (31.2)	15/80 (18.8)
Age, mean (SD), y	47.78 (13.28)	46.15 (13.03)
WRAT Reading grade level, mean (SD)	11.8 (2.2)	11.7 (2.3)

Abbreviations: CCBT, computer-assisted cognitive behavior therapy; TAU, treatment as usual; WRAT, Wide Range Achievement Test.

^a^ Current psychotherapy indicates that patient reported receiving current psychotherapy other than CCBT.

^b^ Antidepressant medication indicates that patient reported current use of antidepressant.

**Figure.**
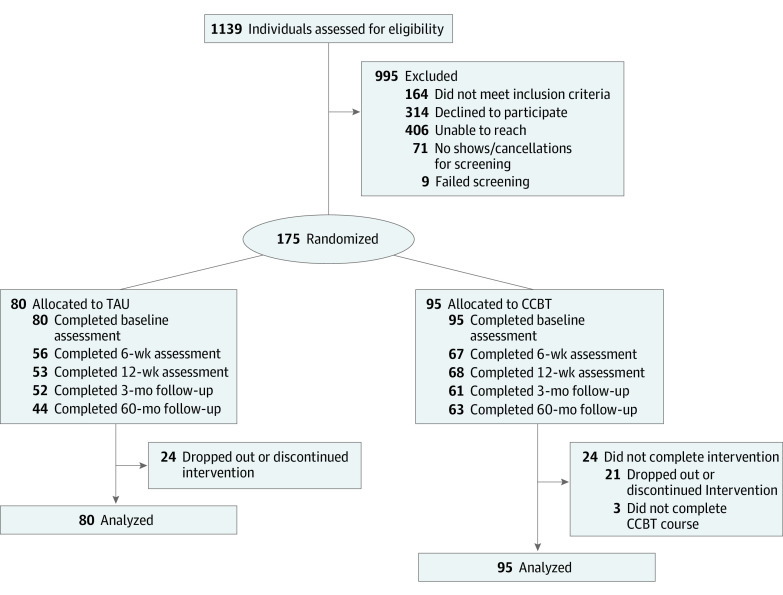
Study Flowchart CCBT indicates computer-assisted cognitive behavior therapy; TAU, treatment as usual.

### Primary Outcome

The results from the ITT analysis are presented in Table 2. PHQ-9 scores decreased over time in both treatments; however, there were larger differences post treatment for the CCBT group vs the TAU group (mean difference, −2.5; 95% CI, −4.5 to −0.9; *P* = .005; *d* = −0.42; *P* = .005), and these effects continued at the 3-month (mean difference, −2.3; 95% CI, −4.5 to −0.8; *P* = .006; *d* = −0.38; *P* = .007) and 6-month (mean difference, −3.2; 95% CI, −4.5 to −0.76; *P* = .007; *d* = −0.52; *P* = .005) follow-ups. The effect sizes were in the moderate range.

**Table 2.** CCBT vs TAU: Intention-to-Treat Effect Sizes and Mean Ratings for Outcome Measures

Group	Baseline	12 wk				3-mo Follow-up				6-mo Follow-up			
		Mean score (95% CI)	Mean difference	Cohen *d*	*P *value	Mean score (95% CI)	Mean difference	Cohen *d*	*P *value	Mean score (95% CI)	Mean difference	Cohen *d*	*P *value
**PHQ-9**													
CCBT	16.1 (14.9 to 17.3)	8.6 (7.4 to 9.8)	−2.5	−0.46	.005	8.8 (7.3 to 10.2)	−2.3	−0.38	.006	9.4 (7.9 to 10.9)	−3.2	−0.52	.01
TAU	16.2 (14.9 to 17.6)	11.1 (9.6 to 12.6)				11.1 (9.7 to 12.4)				12.6 (10.8 to 14.4)			
**ATQ**													
CCBT	87.9 (81.8 to 94.1)	65.8 (59.3 to 72.4)	−13.5	−0.46	.009	67.6 (60.6 to 74.6)	−8.4	−0.29	.01	69.1 (61.9 to 76.3)	−10.3	−0.35	.04
TAU	86.7 (80.7 to 92.6)	79.3 (73.2 to 85.5)				76.0 (69.0 to 83.0)				79.4 (71.8 to 86.9)			
**GAD-7**													
CCBT	12.3 (11.1 to 13.5)	7.1 (5.9 to 8.3)	−2.8	−0.47	.005	8.0 (6.6 to 9.8)	−1.9	−0.32	.002	8.3 (6.8 to 9.8)	−1.6	−0.28	.23
TAU	12.4 (11.2 to 13.7)	9.9 (8.6 to 11.2)				9.9 (8.9 to 11.7)				9.9 (8.2 to 11.7)			
**SWLS**													
CCBT	14.2 (13.0 to 15.5)	17.9 (16.7 to 19.0)	3.3	0.49	.007	18.3 (16.9 to 19.7)	2.6	0.39	.003	17.7 (16.2 to 19.3)	2.9	0.43	.02
TAU	13.4 (11.9 to 14.7)	14.6 (12.7 to 16.5)				15.7 (13.9 to 17.4)				14.8 (13.0 to 16.5)			

Abbreviations: ATQ, Automatic Thoughts Questionnaire; CCBT, computer-assisted cognitive behavior therapy; GAD-7, Generalized Anxiety Disorder–7; PHQ-9, Patient Health Questionnaire–9; SWLS, Satisfaction With Life Scale; TAU, treatment as usual.

### Treatment Response and Remission Rates

Using ITT principles, response and remission rates were calculated at 12 weeks and at 3 and 6 months (eTable 1 in Supplement 2). The rates of response and remission were significantly higher in the CCBT group compared with the TAU group at all measurement points. For example, at post treatment, the response rates were 58.4% (95% CI, 46.4-70.4%) for CCBT and 33.1% (95% CI, 20.7%-45.5%) for TAU. The remission rates were 27.3% (95% CI, 16.4%-38.2%) and 12.0% (95% CI, 3.3%- 20.7%), respectively.

### Secondary Outcomes

There were significant differences in effect sizes in the ITT analysis for the GAD-7, ATQ, and SWLS favoring CCBT vs TAU at all time points, except for the GAD-7 at 6 months (Table 2). At post treatment, patients in the CCBT group had significantly lower ATQ scores (mean difference, −13.5; 95% CI, −20.3 to −3.1; *P* = .009), lower GAD-7 (anxiety) ratings (mean difference, −2.8; 95% CI, −3.8 to −0.7; *P* = .005), and higher SWLS (quality of life) scores (mean difference, 3.3; 95% CI, 0.8 to 5.1; *P* = .007).

### Treatment Completion and Missing Data

The treatment completion rate was 74.7% for CCBT (71 patients). Dropout rates were 22.1% for CCBT (21 patients) and 30.0% for TAU (24 patients). Attrition rates for completion of measures ranged from 28% to 35% for CCBT and from 34% to 45% for TAU across time points (Figure). The type of missing data (ie, complete, intermittent, termination) was not associated with baseline depressive symptoms (*b* = 0.431; SE, 0.619; 95% CI, −0.769 to 1.63; *P* = .49), linear change in depressive symptoms (*b* = 0.064; SE, 0.799; 95% CI, −1.486 to 1.614, *P* = .94), quadratic change (*b* = −0.012; SE, 0.241; 95% CI, −0.479 to 0.455; *P* = .96), or treatment condition (*b* = −0.004; SE, 0.028; 95% CI, −0.058 to 0.050; *P* = .89).

### Factors Associated With Outcomes

Several potential factors associated with CCBT outcome, including baseline PHQ-9 and GAD-7 scores, completion of GDA modules, reading level, education, and antidepressant use were assessed. The number of GDA modules completed was found to be associated with greater symptomatic improvement (estimate, −0.85; 95% CI, −1.49 to −0.22; *P* = .009), but none of the other factors were statistically significant (eTable 2 in Supplement 2).

### Adverse Events

One adverse event was observed. A patient who received TAU died by suicide 4 months after completing a 3-month follow-up, in which no suicidal thoughts were reported. This patient did not complete the 6-month assessment. The study sponsor concurred with our opinion that the death was not related to study procedures. One patient in CCBT dropped out after receiving a computer message about software corruption. However, an investigation found no evidence of compromise of security of the GDA program, and we concluded this was not an adverse event. No other negative reactions to CCBT or TAU were reported.

### CCBT Use

eTable 3 in Supplement 2 displays information about participants’ use of GDA modules. Module completion rates ranged from 89.4% to 44.1%, with lower rates near the end of the program.

The mean (SD) number of clinician sessions completed was 8.83 (3.13) of 12 possible sessions. The mean (SD) amount of telephone support time per session was 17.4 (6.3) minutes. The mean amount of time for emails and texts per patient was 0.24 and 0.90 minutes, respectively. The mean total time for treatment delivery was 18.54 minutes per session and 163.7 minutes for the entire course of treatment. Additional mean technical support time related to questions about using the program was 2.7 minutes.

### Satisfaction Evaluation

Ratings on the Client Satisfaction Questionnaire-8 indicated that CCBT was associated with higher satisfaction with treatment than TAU at all time points. For example, at 12 weeks, the effect size (Cohen *d*) was 1.19 (*P* < .001) (eTable 4 in Supplement 2).

## Discussion

Results of this study show that treatment for depression in primary care can be enhanced by the addition of CCBT to TAU. After 12 weeks of acute treatment, CCBT significantly outperformed TAU in reducing PHQ-9 scores; these positive results were maintained over the 3- and 6-month follow-up intervals. Remission rates were more than double for CCBT compared with TAU at all time points. The between-group ITT effect sizes on the PHQ-9 were in the moderate range, comparable with those reported for other studies of clinician-supported treatment in a meta-analysis of CCBT for depression in primary care settings.^18^

The current study differed from earlier investigations by including patients without computer or internet access and also including substantial numbers of patients with low income, lack of college education, and low levels of reading proficiency. Income of less than $30 000 per year was reported by 61.5% of patients, while 74.3% were not college graduates; and 10.6% had reading test scores of less than ninth grade proficiency—a criterion used to exclude patients from participation in a previous study.^39^ Specific methods were used to widen the economic and educational diversity of patients who might be able to benefit from CCBT (ie, choice of study sites with significant numbers of patients with lower incomes and education, provision of low-cost loaner computers at no charge to patients who needed them). The method of delivering CCBT was designed to maximize convenience for patients. Clinician support was delivered via telephone with email or text communication as desired, and the total amount of time spent with a clinician was less than 3 hours for the entire course of treatment. Thus, we believe that the CCBT method described here has potential for reducing barriers to receiving effective psychotherapy for depression in primary care.

CCBT also produced positive results for negative cognitions, anxiety, and quality of life; and computer-assisted treatment received higher favorability ratings from patients than TAU. Together, these results indicate that CCBT for depression has widespread favorable outcomes.

The effect sizes reported here are somewhat lower than those observed in meta-analyses that included investigations in non–primary care settings.^18,29^ The mean effect size for clinician-supported CCBT in a meta-analysis of 40 such studies was 0.67,^29^ whereas the posttreatment effect size observed here was 0.42. The reasons for possible lower effectiveness in primary care patients are unknown.^18^ However, it has been suggested that study of a clinical population (vs internet recruitment) and presence of medical comorbidities could negatively affect outcome.^18^ Future investigations could compare different settings, demographic characteristics, and treatment methods to better understand the optimal way of engaging and helping patients to benefit from CCBT.

### Limitations

This study had several limitations, including the use of TAU as a control group. Without other comparators, such as standard CBT or TAU plus the amount of clinician support offered here (without GDA), it is impossible to know whether the CCBT method would be as effective as traditional therapies or other control treatments. However, many studies of CCBT vs standard CBT have shown no significant differences in outcome.^38,39,52^ CCBT with the GDA program has been shown to be as effective as standard, face-to-face CBT in 2 previous randomized clinical trials.^38,39^ Although other treatments, such as antidepressants and nonstudy psychotherapy, were not controlled in this study, there were no differences found between CCBT and TAU in use of these treatments. The study was not powered sufficiently to examine the relative effectiveness of CCBT in patients with disadvantages, such as lack of computer access or lower levels of education, nor was it powered to come to firm conclusions about the influence of factors associated with outcomes. The small number of treatment sites is another limitation. The treatment completion rate of 74.7% in CCBT was less than ideal, yet it was higher than the mean completion rate of 58.3% in a recent meta-analysis of CCBT for depression.^29^ Missing values from patients in the CCBT and TAU groups who did not complete outcome measures is an additional limitation. It is unknown whether differences in missing values were random effects, but the analysis of missing values found no significant associations with treatment outcome. Although only 1 adverse event was reported, we did not include a scale to measure potential adverse effects of CCBT.

Other limitations could be addressed in future studies. The GDA program used in this investigation required a desktop, laptop, or notebook computer. However, GDA is now available as a mobile application. At the time this study was designed, telemedicine was not used widely or covered by insurance plans. Subsequent developments in telemedicine influenced by the COVID-19 pandemic suggest that using telemedicine for clinician support in CCBT would be feasible and advantageous.

## Conclusions

The findings of this randomized clinical trial suggest that CCBT with a modest amount of clinician support has potential for wider-spread implementation as an effective, acceptable, and efficient treatment for depression in primary care. The method of CCBT described here may be useful in primary care patients with depression who have low levels of income, education, or reading proficiency as well as in those who lack internet access.

## Notes

Supplement 1.
Trial Protocol

Supplement 2. **eAppendix.** Criteria for Defining Dropout and Treatment Completer Status **eTable 1.** CCBT vs TAU: ITT Response and Remission Rates **eTable 2. **Factors Associated With Symptomatic Improvement: Analysis for Patients Who Received CCBT **eTable 3.** Time Spent per Module and Completion Rate for Good Days Ahead **eTable 4.** Mean Client Satisfaction Questionnaire–8 Scores: Patient Satisfaction for CCBT and TAU

Supplement 3.
Data Sharing Statement
