# Supplementary material for: Effect of Computer-Assisted Cognitive Behavior Therapy vs Usual Care on Depression Among Adults in Primary Care: A Randomized Clinical Trial
Source: JAMA Netw Open. 2022 Feb 10;5(2):e2146716. doi: 10.1001/jamanetworkopen.2021.46716 (PMC8832170; doi:10.1001/jamanetworkopen.2021.46716)
Supplement: Supplement 1. — Trial Protocol [file jamanetwopen-e2146716-s001.pdf]

RESEARCH STRATEGY

Dissemination of Computer-assisted Cognitive-behavior Therapy for Depression in Primary Care

RESEARCH PROCEDURE/STUDY PROTOCOL

Overall Design: A randomized comparison group design is proposed. Substantial changes from our earlier studies in mental health settings are made for dissemination into primary care. Patients in University of Louisville primary care clinics with significant levels of depression (defined by Patient Health Questionnaire [PHQ-9] [Kroenke et al., 2001] scores of 10 or above) will be assigned to one of two conditions: 1) CCBT with telephonic and/or e-mail support + treatment as usual (TAU), or 2) TAU. 120 participants will be randomly assigned to each group for a total sample size of 240. Participants will be assessed five times: baseline, mid- and immediately post-intervention (12 weeks); then at 3 and 6 months following intervention. A cutoff score of 10 on the PHQ-9, a commonly used rating scale in primary care, will be used for inclusion instead of a diagnosis of major depressive disorder (MDD) in this dissemination focused investigation. A cut-off score of 10 or above on the PHQ-9 was chosen because scores of 10 or above indicate at least mild to moderate levels of depression and are consistent with DSM-IV diagnoses of major depressive disorder (MDD), dysthymia, and minor depression (Kroenke et al., 2001). Although we will determine psychiatric diagnoses using the Mini-International Neuropsychiatric Interview (MINI; Sheehan et al., 1998) for purposes of exploring possible predictors, moderators, and mediators, we think that including a broader group of patients with significant depressive symptoms (PHQ-9 of 10 or above) will do a better job of capturing a patient population that primary care clinicians may treat for depression, than narrowing the study group to only those who meet strict diagnostic criteria for MDD. Other treatments such as antidepressants or other psychotherapies will be allowed in TAU, but will be tracked for later comparison among the treatment groups.

Table 1: Hypotheses, Evaluation Methodology, and Statistical Test)

Hypothesis	Evaluation Methodology	Statistical Test
1. Patients who receive CCBT will have high completion rates and comprehension scores compared to benchmark studies of CCBT (Aim 1, Objective A)	CCBT program completion rates and comprehension scores	Benchmark strategy
2. Patients who receive CCBT will report significantly better satisfaction with treatment than those who receive TAU (Aim 1, Objective A)	CSQ-8	Latent growth curve analysis
3. Patients who receive CCBT will demonstrate lower depressive symptomology and greater reduction in negative automatic thoughts post and follow-up than those who receive TAU (Aim 1, Objective B)	PHQ-9, ATQ	Same as #2
4. Patients who receive CCBT will report better quality of life at post and follow-up than those who receive TAU (Aim 1, Objective B)	SWLS	Same as #2
5. Cost-benefit analysis will favor CCBT over TAU (Aim 2, Objectives A, B, & C)	(i) Direct and indirect costs: Medical care utilization cost (including visits to healthcare providers), care coordinator time, antidepressants, lost work days. Data sources: CSRI and insurance plans.<br>(ii) Intervention cost: CCBT program cost<br>(iii) Health benefit: SF-12	Regression (w/bootstrap) to test difference between CCBT and TAU
6. Exploratory analysis: Anxiety disorder diagnosis and severity are moderators of outcomes (AIM 1, Objective C)	GAD-7, MINI	Analyses in hypotheses 2-4 will be repeated with anxiety diagnosis and severity as moderators

7. Exploratory analysis: Patients in CCBT will have greater expectations about change as compared to patients in the TAU condition and this will partially account for outcome differential (AIM 1, Objective C)	PAES	The analyses for hypotheses 2-4 will be repeated with PAES as a mediator
8. Exploratory analysis: Program completion/comprehension scores will be predictors of outcomes (AIM 1, Objective C)	CCBT program completion rates and comprehension scores	The analyses for hypotheses 2-4 will be repeated with CCBT program completion rates and comprehension scores as predictors with only the individuals in the CCBT condition
9. Exploratory analysis: Educational level, diagnoses on MINI, reading level on WRAT, and antidepressant use will be predictors of outcomes	Patient educational level, depressive diagnosis (e.g., MDD based on MINI), reading level on WRAT, and medication use	Analyses for hypotheses 2-4 will be repeated with patient measures as moderators

Referral to Study: Clinicians who practice at the primary care practice sites will receive a brief orientation to the study and educational program on detection of depression and treatment with CCBT (See *Orientation and Education of Clinicians*), after which they will be asked to refer patients for consideration for the investigation. Also, posters and fliers about the study will be available in clinic waiting rooms, and messages about the study will be posted on patient portals for the electronic medical record used at these facilities. In addition, a partial waiver of consent was approved by the University of Louisville IRB for investigators to have access to patient diagnoses and to contact patients with depression regarding possible participation in the study. The study care coordinator will be at the clinical sites to encourage referrals.

Diagnostic Evaluation/ Exclusion Criteria: Patients referred for consideration for the study will be screened by the research associate (RA) with the PHQ-9, the Columbia Suicide Severity Rating Scale (CSSRS, Posner et al., 2003), Wide Range Achievement Test (WRAT) reading test, and a brief questionnaire, including the MINI (Mini Neurodiagnostic Inventory) that will check for exclusion criteria. If the patient meets any of exclusion criteria that do not require a diagnostic evaluation on the MINI, they will not be invited to participate in the diagnostic evaluation with a MINI. Exclusion criteria are: 1) PHQ-9 below 10; 2) refusal to provide informed consent; 3) age 17 or below; 4) patient reports inability to read English text on computer screen; 5) significant suicidal thoughts, intent, plan, or behavior reported on CSSRS;* 6) severe or poorly controlled medical disorders that would interfere with participation in CCBT (e.g., liver failure, terminal cancer); 7) dementia or other organic brain disorders that would prevent participation in CCBT; 8) WRAT reading level of less than 9th grade; 9) diagnosis on MINI of any psychotic disorder or bipolar disorder. Because this study focuses on dissemination of CCBT, other psychiatric diagnoses such as anxiety disorders, eating disorders, or personality disorders will not be exclusion criteria. However, all subjects will participate in an MINI interview before being randomized. The MINI interview, conducted by the research associate, will be used to obtain accurate diagnoses for purposes of study exclusion and for exploring possible predictors, moderators, or modifiers of treatment effectiveness. Patients who have significant suicidal thoughts, intent, plan, or behavior reported on the CSSRS will be referred for urgent evaluation and treatment at University of Louisville facilities or elsewhere, in addition to provision of a 24-hour emergency phone number. Those who report imminent intent and plan for suicide will be referred directly to the Emergency Psychiatry Service at University of Louisville Hospital. The preceptor in primary care will also be notified.
*Defined by answering yes to any of these questions “Have you been thinking about how you might kill yourself,” “Have you had some intention on acting on these thoughts,” “Have you started to work out details of how to kill yourself,” “Do you intend to carry out this plan,” or reports of any recent suicide attempts.

Informed consent and IRB approval: The study has been approved by the University of Louisville IRB, and informed consent will be obtained. See *Protection of Human Subjects* in attached document.

Randomization: At the end of the assessment phase, a web-based randomization procedure will be implemented to balance the baseline characteristics across the two treatment conditions.

Study Therapies – CCBT: For this application in primary care, CCBT will be substantially modified from earlier studies to provide a practical method that can be replicated in non-research settings, supported by current federal and private health insurance plans, and will encourage wide-spread dissemination. Instead of face-to-face support from a cognitive-behavior therapist, clinician support will be provided by a care coordinator or masters level psychotherapist by telephone and/or e-mail or text messages (based on patient preference) on a weekly basis. Telephone delivery of standard CBT in primary care has previously been shown to be an acceptable and effective method and a way to surmount barriers to accessing psychotherapy in primary care patients (Mohr et al., 2012), and telephone and/or e-mail mediated support has been shown to be effective in other studies of CCBT. Telephone and/or e-mail or text support will be scheduled for up to 20 minutes weekly. Actual support time spent by the care coordinator will be monitored and recorded. Coaching sessions by the care coordinator/therapist will be audio recorded and reviewed by investigators for quality control of delivery of the coaching component of treatment. Ratings on the Cognitive Therapy Scale and an adherence checklist will be made to assess competence and adherence.

The “Good Days Ahead” software has 9 lessons that cover the core elements of CBT for depression. Patients will be encouraged to complete all lessons during the 12 weeks of active treatment. The recommended pacing will be: first two lessons of GDA in week one, one of the remaining 7 lessons weekly during weeks 2-8, and use GDA weekly for review and skill consolidation during the last 4 weeks of the 12-week protocol (See *Appendix* for outline of CCBT manual and detailed schedule of lessons and lesson content). However, flexibility in pacing and completion of lesson content will be supported by the care coordinator.

Study Therapies – Treatment as Usual (TAU): Patients assigned to the Treatment as Usual (TAU) condition will receive the standard assessment, clinical care, and service referral by primary care physicians in the primary care setting. Patients assigned to CCBT will also receive TAU.

CCBT Program (“Good Days Ahead”): Drs. Jesse Wright and Aaron Beck, content authors of “Good Days Ahead” (GDA, J Wright, A Wright, & Beck, 2011), are widely recognized as authorities on CBT. Computer programmers and engineers at Empower Interactive, Inc. developed the current online edition of GDA. An overarching principle guiding development of GDA is to provide the highest possible level of patient security. Other specific principles that guided the development of GDA were: 1) be cognizant of problems with concentration, learning, and sustaining effort in depression; 2) use multimedia, especially video and audio, to engage the user and model cognitive CBT strategies; 3) use an experienced cognitive therapist as an online narrator/coach; 4) provide high levels of interactivity; 5) use assessments and self-ratings at multiple points in the program to encourage cognitive processing and learning; 6) promote skill acquisition through varied exercises; 7) utilize appropriate feedback to stimulate user involvement; 8) produce a program that can be used by a wide variety of persons, including those with no previous computer experience; 9) include diverse racial, ethnic, age, and cultural backgrounds in characters appearing in the program; 10) utilize automatic e-mail reminders to assist patients in staying engaged with the program. GDA has been used at multiple universities, military facilities, and other treatment centers. The program has run smoothly without significant technical problems or reports of adverse effects.

GDA includes a series of 9 lessons that help patients build skills in understanding and using CBT to: 1) identify and modify automatic thoughts; 2) use behavioral activation and other behavioral methods; 3) identify and modify schemas (core beliefs); 4) use effective coping strategies; and 5) other core CBT methods. In each lesson, a narrator orients the user to lesson material and explains core concepts of CBT. Videos of a main character who is experiencing depression and anxiety are used to show how CBT skills can help people overcome their problems. After patients view these brief videos and are coached on use of CBT methods, they are engaged in a series of exercises to put the skills to work in their own lives. Mood ratings are completed each time GDA is used. Data from checklists, patient generated records of automatic thoughts and other CBT targets for change, self-help exercises, and mood ratings are stored and made available in easy to read format. Clinicians can access these records and reports through a clinician portal to help guide treatment with CCBT.

GDA is HIPAA compliant, including such features as secure user passwords, encryption of all data both "in transit" and "at rest," and regularly scheduled backups.

Previous studies have found that the core methods of CBT and CCBT are accepted by primary care patients and do not require modification for application in a primary care setting (Proudfoot et al., 2004; Mohr et al., 2012). To further assess the suitability of “Good Days Ahead” for primary care patients, physicians at the Cardinal Station and Newburg clinical sites reviewed the “Good Days Ahead” software and did not recommend any changes for the primary care setting other than a possible audio track feature (text on each page is read to patient if desired) for patients with lower reading or comprehension levels. However, this addition to Good Days Ahead was not possible, and lower levels of reading level (less than 9th grade) excluded patients from participation. Our efforts to provide access to “Good Days Ahead” (onsite computers and loaner computer notebooks for those who do not have access at home, lists of public areas (e.g., libraries, community centers, coffee shops) that have WiFi hot spots and/or computer access, and support from City of Louisville in providing access [See letter from mayor of Louisville]), should make our CCBT method well suited for dissemination into primary care.

Setting: The study settings will be the Department of Family and Geriatric Medicine at the University of Louisville (Cardinal Station, Newburg, and T.J. Sampson Medical Centers) and the Department of Internal Medicine - where faculty and residents care for patients in a private practice environment.

Measures: Data for the study will be obtained from the initial diagnostic assessment and self-report surveys. Data collection instruments and details on validity and reliability are provided in Appendices. See *Data Collection* for method of administration. Instruments were selected based upon their: 1) appropriateness for the target population; and 2) strong psychometric properties (reliability and validity). See Table 2 for measurement points.

CCBT Program Completion and Satisfaction Rates. The GDA software has built in data collection and reporting features to provide these data.

Patient Satisfaction. We will utilize the Client Satisfaction Questionnaire-8 (CSQ-8; Larsen et al., 1979) - an eight-item questionnaire that measures patient satisfaction with mental health services.

Depressive Symptom Severity. We selected the PHQ-9 (Kroenke et al., 2001) as the measure for depression severity because this scale was originally developed for use in primary care, was validated in large samples of medical patients, assesses all 9 DSM-IV symptoms of depression, is easily taken and scored, is a self-rated instrument, and is currently being used widely in primary care. We reasoned that a dissemination-focused project should utilize a self-report depression rating that avoids the special training and costs associated with independent, blind ratings by clinicians.

Cognitions. Common to the study of mental health services utilizing CBT and CCBT, we are interested in specific changes in cognitive styles. Thus, we will utilize the Automatic Thoughts Questionnaire (ATQ; Hollon and Kendall, 1980), a 30-item measure that assesses negative automatic thoughts. We will also use a brief questionnaire, the Computer Attitudes Questionnaire (CAQ) to assess cognitions about using a computer program as part of treatment.

Quality of Life. Patients will complete the Satisfaction with Life Scale (SWLS) at intake and subsequent evaluations. Domains of measurement include work, home, social, and leisure functioning.

Cost-Effectiveness Analysis (CEA). CEA will evaluate the expected costs and expected benefit of CCBT compared to TAU from a societal perspective. Cost data will include (i) healthcare utilization cost, (ii) intervention cost, and (iii) health benefit assessment. Healthcare utilization and lost productivity cost will be recorded using the Client Service Receipt Inventory (CSRI; Beecham & Knapp, 2001) which documents use of health care services and medications, in addition to days off work. We will also utilize claims data on health care utilization and costs supplied by the major insurer (Passport Health Plan, Medicaid insurer, see letter from Dr. Houghland, Chief Medical Officer), University of Louisville Physicians (see letter from Becky Lamb, Vice President), and additional insurers who will supply these data. If the study is funded, we will attempt to secure claims data from all insurers of 5% or more of patients treated at the primary care sites. Thus, CEA will utilize both patient-reported CSRI data and insurance claims data to estimate patient-level costs. Intervention costs of providing CCBT and TAU will be calculated by determining costs of visits to clinicians, support from the care coordinator, and use of GDA ($100 per patient for software use). Software for GDA will be provided free for this study, but an estimated cost of $100 per patient for computation of cost-effectiveness is based on commercial distribution cost of this software. To estimate the expected benefit of CCBT compared to TAU, we will use the

Short Form Health survey SF-12 which allows the generation of quality adjusted life years (QALYs) (Maruish, M. E. (Ed.). (2012). User's manual for the SF-12v2 Health Survey (3rd ed.). Lincoln, RI: QualityMetric Incorporated.)

Exploration of Possible Predictors, Modifiers, and Moderators. The GAD-7 (Spitzer et al., 2006), a measure of generalized anxiety, will be administered to assess levels of anxiety – a common comorbid symptom in primary care patients with depression (Mergl et al., 2007). Also MINI determined diagnoses will be used for exploration of possible effects of comorbid anxiety disorders and other psychiatric conditions. Additional data to be used for this portion of the analysis will be supplied by built-in components of the GDA software. These include reports on amount of lesson material completed, comprehension scores, and satisfaction ratings.
Frequency & Nature of Contact. We will monitor and record the number and frequency (i.e., number of sessions, length of sessions) and nature of contact (e.g., appointments with primary care physician, phone calls by care coordinator, etc.), in addition to use of antidepressants and other therapies, for both groups.
Case Studies of Dissemination of CCBT. If there are specific cases of dissemination issues (e.g., disabilities, rural implementation, underserved implementation) that would make a significant contribution to the scientific literature on use of CCBT, application will be made to the IRB for approval of interviewing such subjects, and IRB stipulations (e.g., obtaining consent for the interview, providing interview questions for reviews) will be followed.

Table 2: Schedule of Assessments

**Measures**	**Pre**	**Mid-Treatment (6 weeks)**	**Post-Treatment (12 weeks)**	**3 & 6 Mo. Follow-up**
CCBT completion rates and comprehension scores		x	x	
PHQ-9, ATQ, SWLS, CAQ GAD-7	X	x	x	x
CSQ-8		x	x	x
CSRI, SF-12	X		x	x
PAES	X	x	x	

Data Collection: Following the initial diagnostic assessment, performed face-to-face on-site at the primary care setting, other surveys will be completed on-line, for those with Internet access, using survey software that meets the requirements of the IRB and HIPAA for information security. For those with internet access and/or cell phones, a link to each survey will be sent via email and text message. Reminder phone calls, emails, and text messages will also be sent by the research associate to promote completion of surveys. Patients can also complete on-line surveys in the primary care setting where they can access computers for the CCBT intervention or via loaned computer notebooks. Surveys will be linked to the CCBT intervention for the 6 and 12-week data collection points in order to maximize response rate, as subjects will be encouraged to complete these surveys at the end of CCBT sessions while they are already on-line at home or on computers available in the primary care setting. For those who can't, or do not, complete on-line measures, surveys will be administered via paper and pencil format or over the telephone. An individualized plan for survey administration will be established during the initial assessment, and access/survey completion will be carefully monitored so that alternate methods can be utilized if on-line surveys are not completed. Incentives for completion of these surveys will be mailed to subjects following confirmation of survey completion at each data point. Participants will be provided with a financial incentive for completion of the outcome measures for this study ($25 at the 6 and 12-week and 3 and 6-month follow-up assessments; total = $100).

Training and Adherence Monitoring:
Care Coordinator. It is anticipated that the care coordinator or masters level therapist hired for this study will not have specialized training in CBT or CCBT, but will have training as a MSW. In the months 4-6 of the study, Dr. Wright will implement an orientation and training program for the care coordinator for support of CCBT. A CCBT manual will be completed by Dr. Wright in the first three months (See Appendices for outline of manual). This manual will be used to assist the care coordinator in learning core elements of the program, methods of

enhancing patient acceptance and use of CCBT, and use of program data to guide patients. The care coordinator will also be oriented to the basic methods of CBT and will be asked to complete a reading assignment of a core text with video illustrations for CBT (Wright, Basco, and Thase, 2006). Dr. Wright will provide supervision to the care coordinator on use of CCBT. Dr. Girdler will supervise the care coordinator.

Research Associate (RA). The RA will be responsible for recruitment, enrollment, randomization, and data collection. Dr. Eells will supervise the RA to ensure he/she follows the study protocol for data collection and interacts appropriately with subjects. The RA will be responsible for maintaining regular contact with subjects to promote completion of the on-line surveys, as well as administration of survey incentives and procedures related to the consent process. However, the RA will not provide guidance in use of CCBT to patients. This function will be solely performed by the care coordinator.

Orientation and Education of Primary Care Clinicians. During the 6-month start-up period at the beginning of the study, Drs. Wright and Girdler, will develop and implement an orientation and education program on the study for primary care clinicians. Two, hour-long educational sessions will be held prior to initiating treatment with CCBT. These sessions will be recorded on video for use in dissemination to other sites and to use for residents or attending physicians who are not able to attend these sessions or who join the clinical team after the initial 6 months of the study. A brief survey will be administered to clinicians after completion of the orientation and education program to assess their knowledge of treatment of depression and their satisfaction with the orientation and education program. Dr. Girdler will meet with clinicians who start work at the site after the first 6 months of the study to orient them to the investigation and encourage review of the video material. In the first 6 months of the study, Drs. Wright and Girdler will develop a succinct manual, including screen shots from GDA, to be used in orienting clinicians to the study.

Support of CCBT by Primary Care Clinicians: During the orientation and education sessions, it will be explained that the care coordinator will be responsible for providing support to patients using CCBT. Clinicians will not be expected to access data from GDA or to provide specific support for use of CCBT. Because this is a dissemination focused study, and clinicians other than care coordinators may wish to access GDA data, track the patient's progress, and provide encouragement to participate in treatment, access to GDA data will be provided for clinicians who wish to do so. Utilization of GDA data (reported via GDA software) and support time by clinicians (brief questionnaire) other than the care manager will be tracked and reported. Clinicians who wish to do so will be provided access to the lessons of GDA to review lesson material.

STATISTICAL ANALYSIS

Statistical Analyses Overview: All analyses will follow the intention-to-treat principle and will make use of the data of all randomized patients. We will test whether the CCBT condition is feasible, provides better outcomes, and is more cost-effective, as compared to the TAU condition. Latent Growth Curve Modeling (LGCM), used as the main analytical approach, is strongly recommended for assessing change in clinical data as estimates of variation in growth trajectories yield information regarding the reliability of treatment effectiveness that is not available using repeated-measures ANOVA. Estimates of the covariance between the growth factors indicate whether initial base-line status on treatment outcomes is related to rate of improvement during treatment, and provides information about treatment effectiveness for specific individuals such as those with more severe depressed symptoms (Duncan, Duncan, & Strycker, 2006). This analytical approach fits well with the study objectives.

Minimizing and Addressing Problems with Missing Data: The investigators appreciate the importance of complete data and have addressed methods to minimize the amount of missing data when conducting clinical trials (Wisniewski et al., 2006). These methods include creating detailed manuals describing study procedures (both clinical care and data collection), training of research staff, generation of reports to indicate when study activities are due, monitoring the status of patients who have not completed scheduled assessments, and clear and ongoing communication among study staff regarding data collection procedures.

Data Analysis Plan: Baseline demographic and clinical characteristics will be described for the sample overall and within each treatment group. To test if the random treatment assignment was balanced, we will examine the treatments in relationship to demographic, psychiatric, and medical characteristics using the appropriate parametric (e.g., analysis of variance) or nonparametric test (e.g., chi-square). The analyses for each specific hypothesis are now presented.

Hypothesis 1, Feasibility**.** We will compare completion rates and comprehension scores for the CCBT condition to benchmarks from other studies. Benchmarking is a method to compare the effectiveness of

services in natural settings against data from other clinical trials or meta-analyses of clinical trials (Minami et al., 2007; 2008). Accordingly, we will use a benchmarking strategy with comparisons of completion rates and comprehension scores from our current R01 project and data from meta-analyses of CCBT for depression.

Hypothesis 2, Satisfaction with CCBT. We will examine satisfaction with CCBT vs TAU, measured with the CSQ-8, via a latent growth curve analysis via multilevel modeling. Specifically, we will test whether treatment condition was a significant predictor of the intercept (Satisfaction-post) and the slope (rate of change). Specifically, we will conduct a two-level model, wherein time (level 1) is nested within patients (level 2). The level 1 model is:

$$Satisfaction = \pi 0ij + \pi 1ij(time) + e$$

where π_{0ijt} is the intercept for client *i* at time *j*, π_{1ij} is the slope or rate of change for client *i* at time *j*, and *e* is the level 1 error. Note by re-centering the time variable (e.g., 0 = 3-month follow-up) that will change the meaning of the intercept, so we can test differences between CCBT vs. TAU at post, 3 and 6-month follow up. At level 2, we include a random effect for patients' intercept and slope (e.g., suggesting that patients might differ in their satisfaction scores and rates of change). These are reflected as *r0* and *r1*. Further, we included CCBT v. TAU (i.e., treatment effects) as a predictor of the intercept (i.e., $\beta 01$) and slope ($\beta 11$). The level 2 model is:

$$\pi 0 = \beta 00 + \beta 01(CCBTv.TAU) + r0i,$$
$$\pi 1 = \beta 10 + \beta 11(CCBTv.TAU) + r1i.$$

***Hypotheses 3-4, Treatment Outcomes*.** We will use the model described in #2, however, PHQ-9 and ATQ scores will be used as the outcome for hypothesis 3 and QLESQ for hypothesis 4.

***Hypothesis 5, Cost Effectiveness*.** Total cost, including the intervention cost, healthcare utilization costs and lost work days, and QALYs will be compared between the treatment groups using regression models, controlling for baseline differences (Manca et al 2005). The regression coefficient on treatment then represents the difference in mean cost and mean QALYs between groups. Bootstrap methods will be used to produce confidence intervals around the cost and QALY differences due to the likely skewness in the distribution of regression residuals (Hoch *et al* 2002). (Bootstrapping generates a large number of re-samples based on the original sample. Parameters of interest, such as cost and QALY differences, are computed for each resample and this allows appropriate confidence intervals to be generated without making any assumption about the data distribution.) The bootstrap replicates will also be graphically presented on the cost-effectiveness plane with QALYs on the x-axis and cost on the y-axis. If one intervention provides more QALYs but higher costs, then we will calculate incremental cost-effectiveness ratios (ICERs) which will indicate the extra costs incurred to achieve an extra unit of QALY gain.

***Hypothesis 6, Anxiety Effects*.** We will replicate the growth curve models from above with the addition of the main effect and interaction effect of anxiety severity (i.e., GAD-7 and SCID) at level 2.

$$\pi 0 = \beta 00 + \beta 01(CCBTv.TAU) + \beta 02(Anxiety) + \beta 03(CCBTv.TAU * Anxiety) + r0i,$$
$$\pi 1 = \beta 10 + \beta 11(CCBTv.TAU) + \beta 12(Anxiety) + \beta 13(CCBTv.TAU * Anxiety) + r1i.$$

***Hypothesis 7, Program/Patient Effects*.** We will examine whether patient expectations/attitudes, measured with the PAES, mediate the effect of treatment group on outcomes. To do so, we will first examine whether the treatment groups predict differences in mid-treatment expectations/attitudes (assuming that there are not differences at pre). If so, we will calculate the direct and indirect effects of treatment group on outcomes via patient expectations and attitudes. These models are not illustrated here due to space restrictions.

***Hypothesis 8, Program Effects*.** In this analysis, we will only include those patients in the CCBT condition. We will examine whether CCBT completion rate and comprehension scores are significant predictors of outcome and slope. The level 1 equation is consistent with above (see # 2) and the level 2 equation is:

$$\pi 0 = \beta 00 + \beta 01(Completion\ Rate) + \beta 02(Comprehension\ score) + r0i,$$
$$\pi 1 = \beta 10 + \beta 11(Completion\ Rate) + \beta 12(Comprehension\ score) + r1i.$$

***Hypothesis 9, Patient Effects*.** Consistent with hypotheses 6 & 8, we will include patient predictors (i.e., patient education level, diagnoses, and medication use) as main effect predictors of outcome and slope. Further, we will test the interaction effects of these patient effects with treatment groups.

References:

Beecham J, Knapp M: Costing psychiatric interventions. In Thornicroft G (ed) Measuring mental health needs. London, Gaskell, 2001

Duncan TE, Duncan SC, and Stryker LS: An Introduction to Latent Variable Growth Curve Modeling: Concepts, Issues, and Applications (Second Edition.) Lawrence Erlbaum Associates, Mahwah, NJ, 2006

Hoch JS, Briggs AH, and Willan AR: Something old, something new, something borrowed, something blue: a framework for the marriage of health econometrics and cost-effectiveness analysis. Health Economics, 11:415-430, 2002

Hollon SD and Kendall PC: Cognitive self-statements in depression: Development of an automatic thoughts questionnaire. Cognitive Therapy and Research, 4:383-395, 1980

Kroenke K, Spitzer RL, and Williams JBW: The PHQ-9: Validity of a brief depression severity measure. Journal of General Internal Medicine, 16:606-613, 2001

Larsen DL, Attkisson CC, Hargreaves WA, and Nguyen TD: Assessment of client/patient satisfaction: Development of a general scale. Evaluation and Program Planning, 2:197-207, 1979

Manca A, Hawkins N, and Sculpher MJ: Estimating mean QALYs in trial-based cost-effectiveness analysis: the importance of controlling for baseline utility. Health Economics, 14:487-496, 2005

Maruish ME, editor. User's manual for the SF-12v2 health survey. 3rd Edition. Lincoln, RI: Quality Metric Incorporated; 2012

Mergl R, Seidscheck I, Allgaier AK, Moller HJ, Hegerl U, and Henkel V: Depressive, anxiety, and somatoform disorders in primary care: prevalence and recognition. Depression & Anxiety, 24(3):185-95, 2007

Mohr DC, Ho J, Duffecy J, Reifler D, Sokol L, et al: Effect of Telephone-administered vs Face-to-Face Cognitive-Behavioral Therapy on Adherence to Therapy and Depression Outcomes Among Primary Care Patients: A Randomized Trial. Journal of American Medical Association, 307(21):2278-2285, 2012

Posner K, Brent D, Lucas C, Gould M, Stanley B, et al: Columbia-Suicide Severity Rating Scale (C-SSRS). In MB First [Ed.] Standardized Evaluation in Clinical Practice, pp. 103-130, 2003

Proudfoot J, Ryden C, Everitt B, et al: Clinical efficacy of computerized cognitive-behavioral therapy for anxiety and depression in primary care: randomised controlled trial. British Journal of Psychiatry, 185:46-54, 2004

Rush AJ: STAR-D: Lessons Learned and Future Implications. Depression and Anxiety 28:521-524, 2011

Sheehan DV, Lecrubier Y, Sheehan KH, et al: The Mini-International Neuropsychiatric Interview (M.I.N.I.): The Development and Validation of a Structured Diagnostic Psychiatric Interview for DSM-IV and ICD-10. Journal of Clinical Psychiatry, 59(suppl 20):22-33, 1998

Spitzer RL, Kroenke K, Williams JBW, Lowe B: A brief measure for assessing generalized anxiety disorder. Archives of Internal Medicine, 166:1092-1097, 2006

Wisniewski SR, Leon AC, Otto MW, and Trivedi MH: Prevention of Missing Data in Clinical Research Studies. Biological Psychiatry, 59:997-1000, 2006

Wright JH, Wright AS, and Beck AT: Good Days Ahead: Empower Interactive, Inc. San Francisco, CA, 2011

Wright JH, Basco MR, and Thase ME: Learning Cognitive-Behavior Therapy: An Illustrated Guide. American Psychiatric Press, Inc. Washington DC, 2006
