# Supplementary material for: Effect of Computer-Assisted Cognitive Behavior Therapy vs Usual Care on Depression Among Adults in Primary Care: A Randomized Clinical Trial
Source: JAMA Netw Open. 2022 Feb 10;5(2):e2146716. doi: 10.1001/jamanetworkopen.2021.46716 (PMC8832170; doi:10.1001/jamanetworkopen.2021.46716)
Supplement: Supplement 2. — eAppendix. Criteria for Defining Dropout and Treatment Completer Status eTable 1. CCBT vs TAU: ITT Response and Remission Rates eTable 2. Factors Associated With Symptomatic Improvement: Analysis for Patients Who Received CCBT eTable 3. Time Spent per Module and Completion Rate for Good Days Ahead eTable 4. Mean Client Satisfaction Questionnaire–8 Scores: Patient Satisfaction for CCBT and TAU [file jamanetwopen-e2146716-s002.pdf]

Supplemental Online Content

Wright JH, Owen J, Eells TD, et al. Effect of computer-assisted cognitive behavior therapy vs usual care on depression among adults in primary care: a randomized clinical trial. *JAMA Netw Open.* 2022;5(2):e2146716. doi:10.1001/jamanetworkopen.2021.46716

eAppendix. Criteria for Defining Dropout and Treatment Completer Status

eTable 1. CCBT vs TAU: ITT Response and Remission Rates

eTable 2. Factors Associated With Symptomatic Improvement: Analysis for Patients Who Received CCBT

eTable 3. Time Spent per Module and Completion Rate for Good Days Ahead

eTable 4. Mean Client Satisfaction Questionnaire–8 Scores: Patient Satisfaction for CCBT and TAU

This supplemental material has been provided by the authors to give readers additional information about their work.

© 2022 Wright JH et al. *JAMA Network Open.*

eAppendix. Criteria for Defining Dropout and Treatment Completer Status

Prior to the start of the study, we specified criteria for defining dropout and completer status. For CCBT, dropout criteria were: (1) patient's expressed intent to discontinue participation in the study before meeting completer status, (b) no participation or contact in six weeks or more, despite at least weekly attempts at contacting patient, and/or (c) no contact with the social worker delivering support for CCBT and/or use of GDA program by week six. For the TAU condition, the dropout criteria were: (1) not completing two more consecutive assessments time points, and/or (2) not completing the post-assessment within the data collection window (one month). Completer status for CCBT was defined as completing at least 2/3 of the therapy content (6 of 9 lessons in GDA and 9 of 12 phone or email sessions with the therapist).

© 2022 Wright JH et al. *JAMA Network Open.*

eTable 1. CCBT vs TAU: ITT Response and Remission Rates

	12-Weeks		3-Months		6-Months	
	Response	Remission	Response	Remission	Response	Remission
**CCBT**	58.4 (46.4 to 70.4)	27.3 (16.4 to 38.2)	52.9 (40.9 to 64.9)	33.2 (22.1 to 44.3)	47.3 (36.6 to 58.0)	29.3 (18.6 to 40.0)
**TAU**	33.1 (20.7 to 45.5)	12.0 (3.3 to 20.7)	29.1 (17.1 to 41.1)	10.2 (2.1 to 18.3)	27.4 (18.1 to 36.7)	13.6 (4.3 to 22.9)

Note: Percent response and remission are displayed with 95% confidence intervals

© 2022 Wright JH et al. *JAMA Network Open.*

eTable 2. Factors Associated With Symptomatic Improvement: Analysis for Patients Who Received CCBT

Predictor	Estimate (95% CI)	P Value
Baseline PHQ-9	-0.92 (-2.81 to 0.97)	.337
Baseline GAD-7	-0.64 (-2.09 to 1.96)	.950
GDA Completion	-0.85 (-1.49 to -0.22)	.009
Anxiety Disorder Dx	-1.81 (-6.61 to 3.00)	.457
Other Psychotherapy	2.31 (-2.57 to 6.58)	.387
On Antidepressant	2.90 (-1.60 to 7.40)	.204
WRAT(reading level)	-1.56 (-3.75 to 0.63)	.161
Educational Level	0.07 (-1.98 to 2.11)	.950
Income	-0.02 (-2.07 to 2.03)	.986
Age	0.61 (-1.30 to 2.52)	.633

Note: GDA completion = number of modules completed

© 2022 Wright JH et al. *JAMA Network Open.*

eTable 3. Time Spent per Module and Completion Rate for Good Days Ahead

Module #	Time Spent<br>*Minutes: Seconds*	Completed Module
	**M (SD)**	**%**
Module 1	15:05 (6:44)	89.4
Module 2	15:01 (6:54)	85.1
Module 3	15:23 (6:55)	83.0
Module 4	14:19 (7:32)	79.8
Module 5	14:30 (6:17)	78.7
Module 6	14:27 (7:21)	77.7
Module 7	13:53 (7:44)	66.0
Module 8	14:16 (7:42)	54.3
Module 9	14:33 (6:17)	44.1

© 2022 Wright JH et al. *JAMA Network Open.*

eTable 4. Mean Client Satisfaction Questionnaire–8 Scores: Patient Satisfaction for CCBT and TAU

Condition	12 weeks	3-month follow-up	6-month Follow-up
**CCBT**	3.53 (3.42 to 3.64)	3.94 (3.82 to 4.06)	3.38 (3.26 to 3.50)
**TAU**	2.70 (2.54 to 2.86)	2.75 (2.57 to 2.92)	2.76 (2.57 to 2.94)
Effect size<br>P value	1.19<br>< .001	1.70<br>< .001	0.89<br>< .001

Note. Mean scores with 95% confidence intervals; Cohen's *d* effect sizes.

© 2022 Wright JH et al. *JAMA Network Open.*
