# Supplementary material for: Effect of Computer-Assisted Cognitive Behavior Therapy vs Usual Care on Depression Among Adults in Primary Care: A Randomized Clinical Trial
Source: JAMA Netw Open. 2022 Feb 10;5(2):e2146716. doi: 10.1001/jamanetworkopen.2021.46716 (PMC8832170; doi:10.1001/jamanetworkopen.2021.46716)
Supplement: Supplement 3. — Data Sharing Statement [file jamanetwopen-e2146716-s003.pdf]

Data Sharing Statement

Wright. Effect of Computer-Assisted Cognitive Behavior Therapy vs Usual Care on Depression Among Adults in Primary Care. *JAMA Netw Open*. Published February 10, 2022.
doi:10.1001/jamanetworkopen.2021.46716

Data
Data available: Yes
Data types: Deidentified participant data
How to access data: Contact principal investigator, Jesse H. Wright, M.D., Ph.D.
jwright@iglou.com
When available: With publication

Supporting Documents
Document types: None

Additional Information
Who can access the data: Researchers whose proposed use of the data has been approved.
Types of analyses: For any purpose
Mechanisms of data availability: With a signed data use agreement.
